# Gene expression and immune infiltration in melanoma patients with different mutation burden

**DOI:** 10.1186/s12885-021-08083-1

**Published:** 2021-04-09

**Authors:** Liwei Wang, Fu Chen, Rui Liu, Lei Shi, Guosheng Zhao, Zhengjian Yan

**Affiliations:** 1 Cancer Center, The Second Affiliated Hospital of Chongqing Medical University, Chongqing, 400010 China; 2 Unit 32357 of People’s Liberation Army, Pujiang, Sichuan, 611630 China; 3 Department of Orthopedics, The Second Affiliated Hospital of Chongqing Medical University, No.76 Linjiang Road, Yuzhong District, Chongqing, 400010 China

**Keywords:** TCGA, SKCM, TMB, ICB, Survival analysis

## Abstract

**Background:**

Immunotherapy is a vital component in cancer treatment. However, due to the complex genetic bases of cancer, a clear prediction index for efficacy has not been established. Tumor mutation burden (TMB) is one of the essential factors that affect immunotherapeutic efficacies, but it has not been determined whether the mutation is associated with the survival of Skin Cutaneous Melanoma (SKCM) patients. This study aimed at evaluating the correlation between TMB and immune infiltration.

**Methods:**

Somatic mutation profiles (*n* = 467), transcriptome data (*n* = 471), and their clinical information (*n* = 447) of all SKCM samples were downloaded from The Cancer Genome Atlas (TCGA) database. For each sample, TMB was calculated as the number of variants per megabase. Based on K-M survival analysis, they were allocated into the high-TMB and low-TMB groups (the optimal cutoff was determined by the ‘surv_cutpoint’ algorithm of survival R package). Then, Gene ontology (GO) and Gene Set Enrichment Analyses (GSEA) were performed, with immune-associated biological pathways found to be significantly enriched in the low-TMB group. Therefore, immune genes that were differentially expressed between the two groups were evaluated in Cox regression to determine their prognostic values, and a four-gene TMB immune prognostic model (TMB-IP) was constructed.

**Results:**

Elevated TMB levels were associated with better survival outcomes in SKCM patients. Based on the cutoff value in OS analysis, they were divided into high-TMB and low-TMB groups. GSEA revealed that the low-TMB group was associated with immunity while intersection analysis revealed that there were 38 differentially expressed immune-related genes between the two groups. Four TMB-associated immune genes were used to construct a TMB-IP model. The AUC of the ROC curve of this model reached a maximum of 0.75 (95%CI, 0.66–0.85) for OS outcomes. Validation in each clinical subgroup confirmed the efficacy of the model to distinguish between high and low TMB-IP score patients.

**Conclusions:**

In SKCM patients, low TMB was associated with worse survival outcomes and enriched immune-associated pathways. The four TMB-associated immune genes model can effectively distinguish between high and low-risk patients.

**Supplementary Information:**

The online version contains supplementary material available at 10.1186/s12885-021-08083-1.

## Background

Skin Cutaneous Melanoma is a fatal malignant tumor of the skin. Currently, the specific etiology of primary cutaneous melanoma is postulated to be long-term ultraviolet overexposure [1, 2]. Ultraviolet short waves induce DNA damage in skin cells, which leads to inflammation, immunosuppression, and melanoma [3]. Even though SKCM accounts for less than 5% of skin cancers, it has a high mortality rate that is correlated with its high malignancy and invasiveness [4]. Over the past 30 years, the global prevalence of SKCM has been on the rise, and it has been reported that in 2018, global incidences were about 280 thousand new morbidites and over 60 thousand mortalities [5]. In 2019, there were more than 96 thousand new cases of SKCM in the USA alone [6]. In China, the annual number of new SKCM cases is more than 8 thousand [7]. Even though SKCM incidences are increasing, advances in medical technology, especially in targeted therapy, immunotherapy, and chemotherapy to a certain extent, have led to positive survival outcomes for patients and reduced mortality rates. For example, before 2011, the median survival time for metastatic melanoma patients was 9 months, but has now been reported to exceed 2 years [8]**.** These improvements are mainly attributed to small molecule inhibitors (e.g., BRAF inhibitors, MEK inhibitors) [9, 10] and immune checkpoint blockade (ICB) [11–13]. Identification of CTLA-4 and PD-L1/PD-1 antibodies has enhanced advances in tumor immunotherapy [14, 15].

The tumor microenvironment is mainly composed of tumor cells, fibroblasts, immune cells and the extracellular matrix, which significantly affect treatment and survival outcomes. Immunotherapy is closely correlated with immune cells. Elucidating on immune infiltration in the tumor microenvironment is key to improving response rates, and could inform the development of new immunotherapeutic strategies. Melanoma is a tumor with strong immunogenicity. Pathological studies have reported that there is a high number of immune cell infiltration in melanoma tissues and, immunotherapy can directly inhibit tumor progression or even cure tumors [16, 17].

Tumor mutation burden refers to the frequency of mutations in the coding regions of somatic cells (variants per megabase) [18–20]. An increase in TMB is correlated with an increase in tumor antigenicity, which is the premise of the effectiveness of the PD-1/PD-L1 antibody. In several clinical trials, TMB has been shown to be a good predictor of immunotherapeutic efficacy [21–25]. The CheckMate-026 clinical trial, a retrospective analysis, reported that: among NSCLC patients administered with immunotherapy, the remission rate and progression-free survival outcomes of the high-TMB group were significantly better than those of the low-TMB group. Patients exhibiting elevated PD-L1 protein expression with high TMB had the most significant benefits, however, patients with elevated PD-L1 protein expression levels but with low TMB had no significant benefits. CM012 post-test analysis revealed corresponding results [21].

Various public databases, such as TCGA, GEO, cBioPortal, and ICGC among others, are available for researchers. However, few studies have evaluated TMB associated immune infiltration in SKCM. Therefore, this study aimed at evaluating the prognostic value of TMB, and to elucidate on how it is associated with immune infiltration.

## Methods

### Data acquisition

Somatic mutation profiles for 467 SKCM patients were retrieved from the TCGA database (https://portal.gdc.cancer.gov/). The “Maftools” R package [26] was used to represent the mutation situation. Moreover, we downloaded the level 3 transcriptome data for all available SKCM samples (tumor samples, *n* = 471). Corresponding clinical information including sex, age, TNM stages, pathological stage, as well as survival outcomes were also obtained (*n* = 447, Table 1, Supplementary Table S1). Samples with follow-up time of less than 60 days were deleted after which the remaining samples were merged with TMB for survival analysis (Supplementary Table S2). The workflow of this study was illustrated in Supplementary Fig. S1. These data were retrieved from free public databases, and as such, ethical approval was waived.

**Table 1:** Clinical baseline of 447 SKCM patients

Variables	Number (%)
**Vital Status**	
Alive	239 (53.5%)
Dead	208B (46.5%)
**Age**	57.8 ± 15.6
**Sex**	
Male	279 (62.4%)
Female	168 (37.6%)
**Pathological Stage**	
I/II NOS	10 (2.3%)
Stage 0	6 (1.3%)
Stage I	77 (17.2%)
Stage II	131 (29.3%)
Stage III	170 (38.0%)
Stage IV	20 (4.5%)
Unknown	33 (7.4%)
**AJCC-T Stage**	
T0/Tis	30 (6.7%)
T1	42 (9.4%)
T2	76 (17.0%)
T3	88 (19.7%)
T4	144 (32.2%)
TX	42 (9.4%)
Unknown	25 (5.6%)
**AJCC-N Stage**	
N0	222 (49.7%)
N1	73 (16.3%)
N2	49 (11.0%)
N3	55 (12.3%)
NX	31 (6.9%)
Unknown	17 (3.8%)
**AJCC-M Stage**	
M0	402 (89.9%)
M1	21 (4.7%)
Unknown	24 (5.4%)
**Sample Type**	
Primary tumor	95 (21.2%)
Metastatic	352 (78.8%)

### TMB calculation and Kaplan-Meier analysis

TMB refers to the total number of substitutions, insertions, deletions, and mutant genes per megabase in the coding region (exon) of the gene assessed in the tumor tissue. In this study, we determined TMB by dividing the number of variants by the length of exons (38 million) for each sample. TMB and the corresponding survival time of the same sample were merged. Subsequently, Kaplan-Meier (KM) analysis was performed to compare survival outcomes in low- versus high-TMB groups and determined the p of the log-rank test. Moreover, Wilcoxon rank test was performed to assess the difference between two groups of different clinical characteristics while the Kruskal-Wallis test was performed to compare differences among multiple groups.

### Differentially expressed genes and functional pathway analysis

RNA-seq data of SKCM patients were divided into low- and high-TMB groups. The “limma” R package [27] was used to identify DEGs between the two groups (Fold Change [FC] = 1 and False Discovery Rate [FDR] < 0.05)., and the Heatmap plot was drawn using “pheatmap” R package (https://CRAN.R-project.org/package=pheatmap) to indicate the difference. Next, the Entrez ID for every DEG was obtained using the “org. Hs.eg.db” package, after which GO analysis was performed using “clusterProfiler” [28], “enrichplot” and “ggplot2” packages. In addition, GSEA [29] was performed using the TMB level as the phenotype. The “c2.cp.kegg.v7.1.symbols.gmt” was retrieved from the MSigDB database [30], and was set as a baseline gene set. Pathways with FDR < 0.25 were considered enriched. Due to differences in immune pathway enrichment in high versus low TMB groups, we used the “estimate” R package (https://R-Forge.R-project.org/projects/estimate) to calculate immune cell scores for the transcriptome data. From the Immunoscore survival analysis, it was shown that low immune infiltration is associated with poor survival outcomes. Therefore, interactions between DEGs and immune-related genes (ImmPort Private Database) were evaluated. Venn analysis showed that there were 38 intersection genes for 1812 immune genes [31] and 504 DEGs.

### Construction of a TMB-immune prognostic model for hub immune genes

Univariate-lasso-multivariate Cox regression analysis was performed on the 38 intersection genes, from which four hub immune genes were obtained and used to construct the TMB-IP model. Then, we calculated the TMB-IP score for all patients by the coefficients of each gene and divided the SKCM patients into high and low TMB-IP group. ROC curve and K-M analysis were used to assess the predictive value of the TMB-IP score in SKCM. The prognostic efficiency of this model was also tested in each clinical subgroup through K-M survival analysis.

### TIMER database and CIBERSORT algorithm

Mutation types of the hub immune genes with different immune infiltrates in SKCM were assessed according to the “SCNA” module of Tumor Immune Estimation Resource (TIMER) database [32]. Furthermore, “CIBERSORT” algorithm [33] was used to estimate the immune infiltration degree of 22 types of cells in a mixed population of cells based on certain features of gene expression in 22 leukocyte subtypes-LM22. The “pheatmap” package revealed immune cell distributions in the two groups. Then, we used the Wilcoxon rank test to assess disparities in the amounts of immune infiltrates in the low-versus high-TMB groups. The “vioplot” R package was used to determine the *p*-values.

### Determination of prognostic value of immune cells in the TIMER database

Data from the TIMER database were used to perform multivariate Cox analysis of the cells that were involved in immune infiltration, as well as to calculate the hazard ratio (HR; 95%CI). Survival outcomes were evaluated by survival analysis.

### Statistical analysis

R software (Version 3.5.2) was used to conduct all the data analysis in the present study. The optimal cutoff for samples classified into better and worse survival groups was determined by the ‘surv_cutpoint’ algorithm of survival R package.

## Results

### Mutation profiles in SKCM

**Fig. 1:**
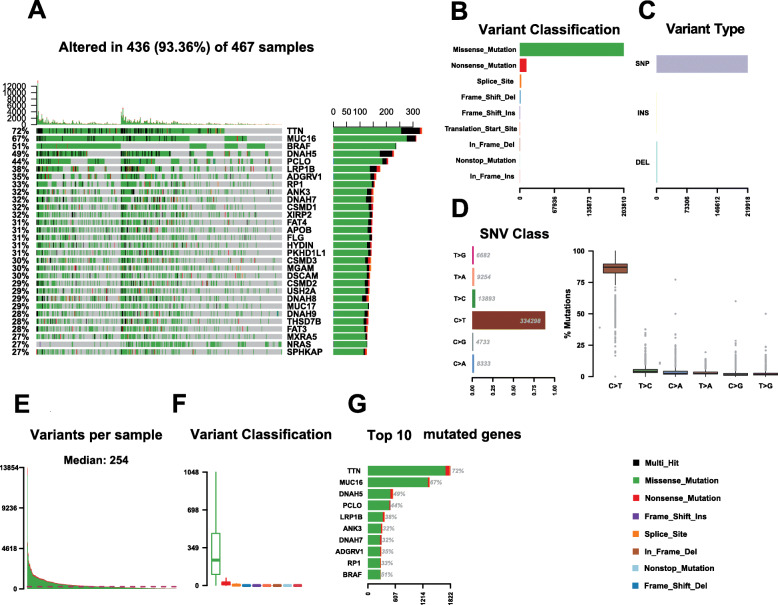
Mutation profile landscape in SKCM samples. **a** Waterfall plot showing the mutation details of every gene in every sample; **b**,**c**,**d** Various types of mutation classified based on different groups; **e**, **f** burden of tumor mutation in particular samples; **g** Top ten mutated genes. Various types of mutations are represented by different color annotations at the right-bottom

Somatic mutation profiles for 467 SKCM patients were retrieved from the TCGA database. According to the mutation data, “Maftools” R package was used to examine the findings. The waterfall plot was used to present the mutation data for every gene in every sample (Fig. 1a). Further, mutations were grouped based on various categories. In the grouping, missense mutations were the most common (Fig. 1b), while deletion/insertion mutaions were common than single nucleotide polymorphisms (SNP) (Fig. 1c). Regarding single nucleotide variants in SKCM, C > T occurred more often (Fig. 1d). In addition, we constructed an SKCM box plot (different colors denote different mutations) to reveal mutation types based on the number of changed bases in every sample (Fig. 1e, f). Figure 1g shows the top ten mutated genes in SKCM, which were TTN (72%), MUC16 (67%), BRAF (51%), DNAH5 (49%), PCLO (44%), LRP1B (38%), ADGRV1 (35%), RP1 (33%), ANK3 (32%) and DNAH7 (32%). Supplementary Fig. S2 shows coincidences, as well as exclusive relationships among the mutated genes (green denotes co-occurrence, whereas red denotes relationships that are mutually exclusive). It is shown that genes with higher mutations in SKCM appear at the same time, and that there are no obvious repulsive genes. The genecloud plot was established to show the frequency of mutations in other genes (Supplementary Fig. S2).

### TMB is correlated with survival outcomes, pathological stage and tumor grade

**Fig. 2:**
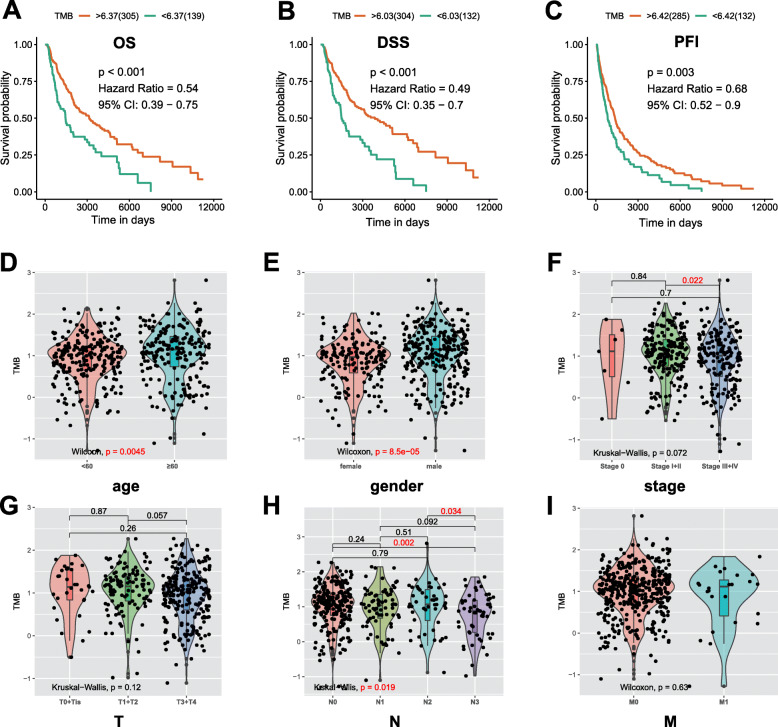
TMB prognosis and its relationship with clinical features. Higher TMB levels correlated with better OS (**a**), better DSS (**b**), and better PFI (**c**); **d**-**i** Wilcoxon and Kruskal-Wallis test in groups of different clinical characteristics

Based on the above mutation data profile, we computed the number of mutation events/million bases as TMB for the 467 samples. The optimal cutoff for samples classified into better and worse survival groups was established by the ‘surv_cutpoint’ algorithm of survival R package, consistent with the recognition that higher TMB enhances immune recognition and leads to better disease outcomes. High-TMB group patients exhibited better OS outcomes (*p* < 0.001, HR = 0.54, 95%CI = 0.39–0.75; Supplementary Table S2, Fig. 2a), better DSS (*p* < 0.001, HR = 0.49, 95%CI = 0.35–0.7; Supplementary Table S2, Fig. 2b) and better PFI (*p* = 0.003, HR = 0.68, 95%CI = 0.52–0.9; Supplementary Table S2, Fig. 2c) than the low-TMB group. Moreover, elevated TMB levels were correlated with advanced age (*p* = 0.0045; Fig. 2d), male (*p* = 8.5e− 05; Fig. 2e), lower tumor pathological stages (stage I + II vs. stage III + IV, *p* = 0.022; Fig. 2f) and lower AJCC-N stages (N0 vs. N3, *p* = 0.002; N2 vs. N3, *p* = 0.034; Fig. 2h). However, there were no significant differences between the associations of TMB and AJCC-T (Fig. 2g) and AJCC-M (Fig. 2i).

### Differential expression analysis between two groups

**Fig. 3:**
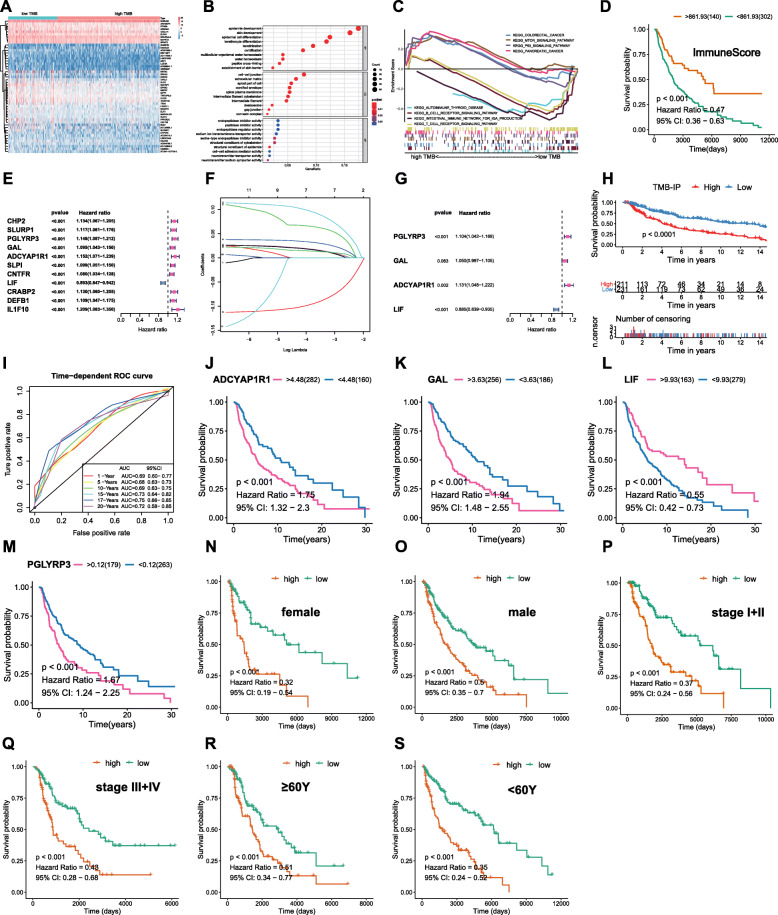
Differentially expression analysis and Construction of TMB-IP. **a** Top 50 DEGs were displayed in heatmap plot; **b** GO results of 504 DEGs; **c** GSEA revealed the top TMB- associated pathways revealed that low-TMB was correlated with immune-related pathways; **d** Survival analysis based on the immunescore; **e**-**g** Univariate-lasso-multivariate Cox regression of 38 immune-related DEGs; **h** Survival analysis based on the TMB-IP score; **i** Multi-year ROC curves based on the TMB-IP score; **j**-**m** Survival analysis of four immune-related hub genes based on their RNA expression levels; **n**-**s** Survival analysis of six clinical subgroups based on high and low TMB-IP grouping

Among the 471 RNA-seq samples, a total of 467 samples were selected from the same source as mutation data. Based on the TMB cutoff value of the OS analysis (cut-off = 6.37, Fig. 2a), we divided the RNA-seq data for 467 tumor samples into low (*n* = 148) and high-TMB (*n* = 319) groups (Fig. 3a). Using the “limma” R package, a total of 504 DEGs were identified between these two transcriptomic data sets with FC = 1 and FDR < 0.05. GO enrichment analysis revealed that the DEGs were mainly involved in epidermis development, skin development, and other biological processes (Fig. 3b). Further, we analyzed the GSEA data of the top TMB-associated items. Figure 3c shows that the low-TMB group was associated with immunity, and was mainly concentrated in autoimmune thyroid disease, B and T cell receptor signaling pathways, as well as intestinal immune network for the production of IGA, while the high-TMB group lacked this function (Supplementary Table S3–4). Given that TMB was associated with immune signature/pathways in SKCM, various analyses were performed on the tumor microenvironment. Using the transcriptome data, the “estimate” R package was used to calculate immunescores for all samples (Non-tumor samples were deleted, OS time over 60 days were merged, *n* = 442, Supplementary Table S5). The score represents the infiltration degree of immune cells in each sample. The higher the score, the more immune cells infiltrated the sample. Immunoscore survival analysis revealed that lower immune infiltration is associated with poor prognosis (*p* < 0.001, HR = 0.47, 95%CI = 0.36–0.63; Fig. 3d). These findings imply that the lower TMB led to immune-associated pathway enrichment while the lower immune infiltrate group was correlated with poor prognosis. Therefore, we evaluated the predictive accuracy of the TMB-associated immune genes. Venn analysis showed that, from 1812 immune genes and 504 DEGs, there were 38 intersecting genes (Supplementary Fig. S3).

### Construction and assessment of TMB-IP in SKCM

Prognostic values of the 38 immune-related DEGs were further evaluated. Data for the 442 SKCM patients were used in univariate-lasso-multivariate Cox regression analysis. A total of 11 genes obtained from the univariate Cox analysis (*p* < 0.001; Fig. 3e) while 7 genes were obtained from lasso regression analysis (PGLYRP3, GAL, ADCYAP1R1, SLPI, CNTFR, LIF, and CRABP2; Fig. 3f). Finally, PGLYRP3, GAL, ADCYAP1R1 and LIF (Fig. 3g) were selected to construct the Tumor Mutation Burden Immune Prognostic model (TMB-IP) using the following formula: TMB-IP score = (PGLYRP3*0.0988045135949462 + GAL*0.0483710471635067 + ADCYAP1R1*0.123387518699318 − LIF*0.121787959868336). We calculated the TMB-IP score for each SKCM patient (Supplementary Table S6). The K-M plot revealed that high TMB-IP score patients exhibited worse survival outcomes (Fig. 3h). Using a cutoff of 0.97080003, patients were divided into high (*n* = 231) and low (*n* = 211) TMB-IP groups. We drew the ROC curves to evaluate the predictive accuracyof the model. The OS outcomes were: AUC = 0.69 (95%CI = 0.60–0.77) for 1-year, 0.68 (95%CI = 0.63–0.73) for 5-years, 0.69 (95%CI = 0.63–0.75) for 10-years, 0.73 (95%CI = 0.64–0.82) for 15-years, 0.75 (95%CI = 0.66–0.85) for 17-years and 0.72 (95%CI = 0.58–0.85) for 20-years (Fig. 3i). Batch survival analysis indicated that these four prognostic hub immune genes were closely correlated with survival outcomes. Suppressed expression levels of ADCYAP1R1 (*p* < 0.001, HR = 1.75, 95%CI = 1.32–2.3; Fig. 3j), GAL (*p* < 0.001, HR = 1.94, 95%CI = 1.48–2.55; Fig. 3k) and PGLYRP3 (*p* < 0.001, HR = 1.67, 95%CI = 1.24–2.25; Fig. 3m) were positively correlated with better prognosis, while suppressed expression levels of LIF (*p* < 0.001, HR = 0.55, 95%CI = 0.42–0.73; Fig. 3l) were negatively correlated with better survival outcomes. Importantly, we further assessed the prognostic efficiency in different clinical subgroups. It was found that high and low TMB-IP score classification criteria can perfectly distinguish the prognostic outcomes for: female (*p* < 0.001, HR = 0.32, 95%CI = 0.19–0.54; Fig. 3n), male (*p* < 0.001, HR = 0.5, 95%CI = 0.35–0.7; Fig. 3o), stage I + II (*p* < 0.001, HR = 0.37, 95%CI = 0.24–0.56; Fig. 3p), stage III + IV (*p* < 0.001, HR = 0.43, 95%CI = 0.28–0.68; Fig. 3q), ≥60Y (*p* < 0.001, HR = 0.51, 95%CI = 0.34–0.77; Fig. 3r) and < 60Y (*p* < 0.001, HR = 0.35, 95%CI = 0.24–0.52; Fig. 3s) patient groups.

### Relationships between mutants and immune infiltrates

**Fig. 4:**
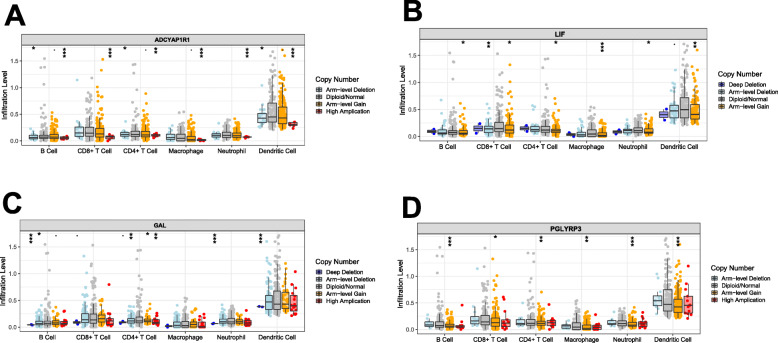
Associations mutants with immune cells infiltration of four hub genes. **a**-**d** Mutants (Arm-level Deletion, Deep Deletion, High Amplication, Arm-level Gain) of four TMB-associated genes exhibited low level of immune cell infiltration compared with Diploid/Normal

The link between the mutants of the 4 hub genes and immune infiltrates in the SKCM microenvironment was assesssed. Based on the findings, immune infiltrate inhibitions, such as CD8+ T cells, dendritic cells, CD4+ T cells, neutrophils, B-cells, and macrophages depended on the type of mutation exhibited by the genes, relative to the levels of immune infiltration in the wild type samples (Fig. 4).

### Variations in the abundance of immune cell infiltration in the low- versus high-TMB groups

**Fig. 5:**
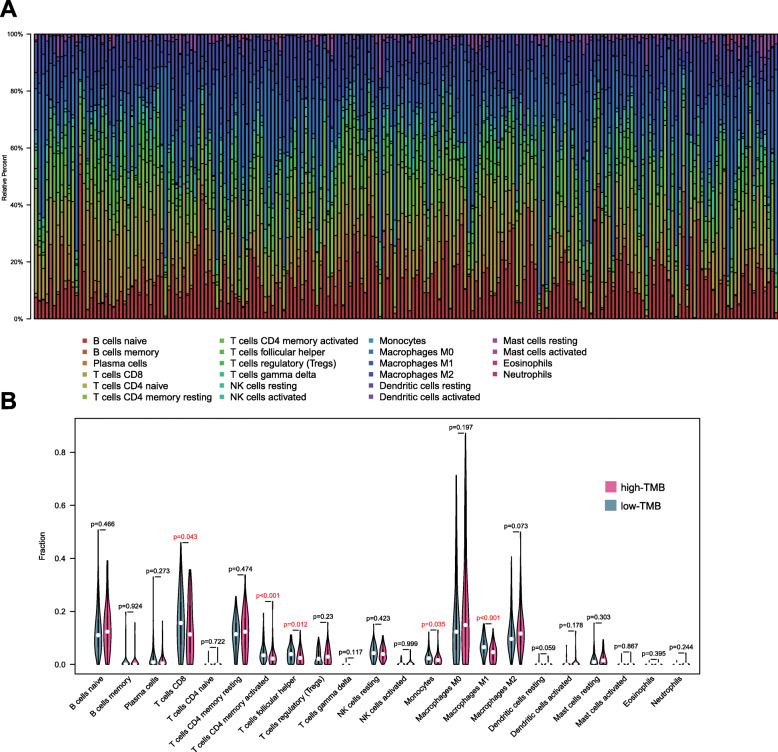
Twenty-two immune fractions in low vs. high-TMB groups. **a** Barplot of the 22 immune fractions denoted by different colors: **b** Wilcoxon test of 22 immune fractions in high- vs. low-TMB group

We have shown that the DEGs negatively impacted on immune pathways and that mutants of the four hub genes were inversely correlated with immune infiltrates. We further compared the various immune fraction profiles in the high- versus low-TMB groups. We used the Voom algorithm in “limma” R package to normalize the transcriptome data for SKCM samples. Samples were filtered at *p* > 0.05 using the “CIBERSORT” R package, and 201 samples (125 low-TMB samples and 76 high-TMB samples) were identified, which were then used to analyze the immune cells (Supplementary Table S7). A box plot was constructed to show specific fractions of 22 immune cells based on “CIBERSORT” algorithm in every SKCM sample (Fig. 5a). These 22 types of immune cells are subclasses of the 6 immune cells in Fig. 4. Wilcoxon rank test revealed that infiltration levels of CD8+ T cells, CD4+ memory activated T cells, follicular helper T cells, monocytes and macrophage M1 cells were reduced in the high-TMB group, relative to those in the low-TMB group (Fig. 5b). Correlation and distribution analysis of the infiltration degree of 22 immune cells in the two groups are shown in Supplementary Fig. S4.

### Association between immune cell infiltration and survival

The Cox regression model was used to determine the association between immune cells and prognosis in SKCM samples (Table 2). In the model, Survival (SKCM) = B cell + CD8 T cell + CD4 T cell + Macrophage + Neutrophil cell + Dendritic cell + ADCYAP1R1 + LIF + GAL + PGLYRP3. Elevated B cell, CD4+ T cell, Macrophage cell infiltrates eas well as elevated expression levels of ADCYAP1R1, GAL, PGLYRP3 were found to be risk factors for SKCM (HR > 1). Additionally, we performed the K-M analysis (Supplementary Fig. S5), where elevated infiltration levels of B cells (*P* = 0), CD8+ T cells (P = 0), Neutrophils (P = 0) and Dendritic cells (P = 0) were positively correlated with better SKCM prognosis, consistent with the result of the survival analysis of the overall infiltration of immune cells in the microenvironment (Fig. 3).

**Table 2:** Multivariate Cox regression analysis of six immune infiltration cells and four immune-related genes

covariates	coef	HR	95%CI_lower	95%CI_uper	*p*.value
B_cell	1.132	3.101	0.095	100.923	0.524
CD8_Tcell	−1.421	0.242	0.02	2.869	0.261
CD4_Tcell	0.089	1.093	0.047	25.157	0.956
Macrophage	1.938	6.944	0.728	66.19	0.092
Neutrophil	−0.53	0.589	0.001	478.663	0.877
Dendritic	−1.378	0.252	0.045	1.428	0.119
ADCYAP1R1	0.244	1.277	0.848	1.923	0.242
LIF	−0.134	0.875	0.805	0.951	0.002
GAL	0.058	1.06	0.978	1.148	0.156
PGLYRP3	0.163	1.177	1.001	1.384	0.049

Rsquare = 0.111 (max possible = 9.93e-01); Likelihood ratio test *p* = 2.94e-07; Wald test *p* = 5.64e-07; Score (logrank) test *p* = 1.82e-07

## Discussion

We analyzed the SKCM-cohort in TCGA. It was found that higher TMB is associated with better survival outcomes and that transcriptomic data for low-TMB patients was enriched in immune-related pathways. Prognostic model construction confirmed that the four immune-related genes can effectively distinguish patients with different prognostic outcomes.

Melanoma, a highly proliferating tumor, originates from melanocytes in the neural crest. It occurs in the skin, intestinal tract, and genital mucosa. Combinations of immunotherapy and targeted therapy has completely changed the treatment of SKCM. In 2013, *Science* ranked tumor immunotherapy at the top of ten scientific breakthroughs [34]. Immunotherapeutic agents targeting CD8+ T-cell surface receptors (CTLA-4 and PD1) have greatly improved patient prognosis [8]. Snyder et al. found that TMB is correlated with clinical benefits of immunotherapy, that is, the greater the number of somatic mutations, the more likely the tumor is to respond to ICB [35–37].

Through integration and unified processing of high-throughput sequencing data for multiple samples from the TCGA database, we established significant mutation genes that may be involved in immune responses of SKCM. It is important to establish molecular markers of immunotherapy to inform individualized treatment of melanoma patients using immunotherapy.

Survival analysis revealed that the high-TMB group patients exhibited better survival outcomes than those in the low-TMB group. Median survival outcomes for the high-TMB group and low-TMB group were 1438 days and 698 days, respectively. Melanoma is a tumor type with a high number of immune cells. Some mutations produce new antigens that are recognized by T cells, which may be one of the reasons for the better survival outcomes of the high-TMB group. Besides, higher TMB correlated with advanced age, male, lower tumor pathological stages, and lower AJCC-N stages. These findings are consistent with those of other studies. Rizvi et al. reported that NSCLC patients with elevated non-synonymous mutation burdens can achieve long-term remission by immunotherapy, which is of paramount importance for PFS [38]. The CheckMate-227 trial, comparing nivolumab+ipilimumab, nivolumab, and chemotherapy, revealed that patients with high TMBs (> 10 mutations/Mb) have the best OS outcomes [39], supporting the validity of TMB-based treatment stratification.

Differential expression analysis and GSEA showed that the low-TMB group was correlated with autoimmune thyroid disease, B-and T-cell receptor signaling pathways, and intestinal immune networks for IGA production. This finding is unexpected and surprising because there is no enrichment of any immune-related pathway in the high-TMB group. GSEA is a method for enriching biological pathways based on hundreds of gene sets. The close relationships between low-TMB and multiple immune-related pathways have been reported, but the specific mechanisms should be further evaluated. Moreover, survival analysis based on immune score of microenvironments indicated that higher levels of immune infiltration are associated with better survival outcomes. Infiltration levels of a variety of immune cells, mainly T cells, can react with tumor or non-tumor antigens, which affects survival outcomes for patients. We also analyzed the effect of each type of immune cell infiltration on survival.

Finally, a four-immune gene prognostic model was developed based on 38 intersecting genes from 1812 immune genes and 504 DEGs, which are greatly useful for survival prediction. Four hub genes were either positively correlated (LIF) or negatively correlated (PGLYRP3, GAL, and DCYAP1R1) with prognosis. The median survival outcomes for high TMB-IP score and low TMB-IP score groups were 804 days and 1490 days, respectively. TMB-IP score is a risk score of the prognostic model. High TMB-IP score means high risk, and it has a relatively shorter median survival outcomes than the low TMB-IP score (low risk) group. Similar findings were obtained in the survival analysis of the six subgroups.

Furthermore, mutants of these hub immune genes were associated with immune infiltrates, such as CD8+ T cells, CD4+ T cells, dendritic cells, macrophages, and B-cells in the SKCM microenvironment, implying that these immune infiltrate cells might be suppressed by mutations. To some extent, this is consistent with the finding of Rosenthal: the absence of new antigens in high immune infiltrated lung cancer suggests that these tumors may evade immune attacks by inhibiting the expression of new antigens [40]. Differences in immune infiltration may affect tumor immune editing and the appearance of neoantigens in tumors. During tumor evolution, tumors with low immune infiltrations exhibit reduced tumor antigen editing, indicating that the historical immune editing or the original clone neoantigen copy is lost. It has been found that immune cell activation in the tumor microenvironment is not the same in different tumor types, even in the same mutation feature, therefore, a specific analysis is needed [41, 42]. We have shown that in SKCM patients, infiltration levels of CD4+ memory activated T cells, CD8+ T cells, follicular helper T cells, Monocytes, and Macrophage M1 cells in the high-TMB group were significantly suppressed. Even though there were no significant differences in other categories of immune cells, the degree of infiltration was not an absolute trend as described above. There were immune cells with higher infiltration levels in the high-TMB group, and specific reasons for this outcome are worthy of further investigations.

In the tumor microenvironment, different immune cells confer different tumor responses. T-helper-1 cells, natural killer cells, M1 phenotype macrophages, and DC1 phenotype dendritic cells are involved in the suppression of tumorigenesis and development, while T-helper-2 cells, M2 macrophages, DC2 dendritic cells, and regulatory T cells (Treg) suppress immune responses [43–45]. Analysis of the unique properties of immune cells in the tumor microenvironment may inform the design of cancer immunotherapy targets. The correlation between lymphocyte infiltration in the tumor microenvironment and immunotherapeutic benefits have been confirmed [46–49]. Higher infiltration levels of B cells, CD8+ T cells, Neutrophils, and Dendritic cells are positively correlated with better SKCM prognosis. Changes in tumor immune microenvironment, tumor gene mutation, and gene regulation may affect tumor evolution and survival outcomes.

However, there are some imperfections. First, we did not carry out experiments (vivo/vitro) to verify the relationship between four immune genes and immune infiltration. Second, the sample size was not adequate to confirm the potential relationship between TMB and prognosis and immune infiltration. It will increase the persuasiveness of the above results if we can further improve these aspects in the future.

## Conclusions

In summary, lower TMB is correlated with worse survival outcomes and immune-related pathways, while higher TMB inhibits immune infiltration in SKCM patients. The four TMB-related immune gene model can effectively differentiate between high and low-risk patients, moreover, mutants of the four hub genes confer lower immune cell infiltration, which should be further validated.

## Supplementary Information

**Additional file 1: Figure S1.** Workflow of this study.

**Additional file 2: Figure S2.** The genecloud plot and the coincidences&exclusive relationship plot among the mutated genes.

**Additional file 3: Figure S3.** Venn analysis of 1812 immune genes and 504 DEGs.

**Additional file 4: Figure S4.** Correlation and distribution analysis of the infiltration degree of 22 immune cells in the high-TMB and low-TMB groups.

**Additional file 5: Figure S5.** K-M analysis based on the infiltration levels.

**Additional file 6: Table S1-S7.**

## Data Availability

The TCGA-SKCM datasets: https://portal.gdc.cancer.gov/; ImmPort Private Database: https://immport.niaid.nih.gov/home; TIMER Database: https://cistrome.shinyapps.io/timer/

## Abbreviations

AUC: Area under the curve
CI: Confidence interval
DEGs: Differentially expressed genes
DSS: Disease Specific Survival
GO: Gene ontology
GSEA: Gene Set Enrichment Analysis
HR: Hazard ratio
ICB: Immunocheck point blockade
OS: Overall Survival
PFI: Progression Free Interval
ROC curve: Receiver operating characteristic curve
SKCM: Skin Cutaneous Melanoma
SNP: Single nucleotide polymorphism
SNV: Single nucleotide variant
TCGA: The Cancer Genome Atlas
TMB: Tumor mutation burden

## References

[1] De La Cruz MM, Abdul Z, Shariff Z. The impact of a skin cancer diagnosis on travel insurance: a sun worshipper's dilemma. Clin Exp Dermatol. 2020. 10.1111/ced.14505 33175406

[2] Siegel JA, Yudkin JS, Craker K, Hwang A, Libby T. Uncapping the bottle: a proposal to allow full-sized sunscreens in carry-on luggage to promote sun protection and prevent skin cancer. J Am Acad Dermatol. 2020. 10.1016/j.jaad.2020.10.066 33129940

[3] Khan AQ, Travers JB, Kemp MG (2018). Roles of UVA radiation and DNA damage responses in melanoma pathogenesis. Environ Mol Mutagen.

[4] Gupta R, Janostiak R, Wajapeyee N. Transcriptional regulators and alterations that drive melanoma initiation and progression. ONCOGENE. 2020;39(48):7093–105. 10.1038/s41388-020-01490-x PMC7695596 33024276

[5] Bray F, Ferlay J, Soerjomataram I, Siegel RL, Torre LA, Jemal A (2018). Global cancer statistics 2018: GLOBOCAN estimates of incidence and mortality worldwide for 36 cancers in 185 countries. CA Cancer J Clin.

[6] Siegel RL, Miller KD, Jemal A (2019). Cancer statistics, 2019. CA Cancer J Clin.

[7] Chen W, Zheng R, Baade PD, Zhang S, Zeng H, Bray F, Jemal A, Yu XQ, He J (2016). Cancer statistics in China, 2015. CA Cancer J Clin.

[8] Luke JJ, Flaherty KT, Ribas A, Long GV (2017). Targeted agents and immunotherapies: optimizing outcomes in melanoma. Nat Rev Clin Oncol.

[9] Schreuer M, Jansen Y, Planken S, Chevolet I, Seremet T, Kruse V, Neyns B (2017). Combination of dabrafenib plus trametinib for BRAF and MEK inhibitor pretreated patients with advanced BRAFV600-mutant melanoma: an open-label, single arm, dual-Centre, phase 2 clinical trial. Lancet Oncol.

[10] Long GV, Hauschild A, Santinami M, Atkinson V, Mandalà M, Chiarion-Sileni V, Larkin J, Nyakas M, Dutriaux C, Haydon A, Robert C, Mortier L, Schachter J, Schadendorf D, Lesimple T, Plummer R, Ji R, Zhang P, Mookerjee B, Legos J, Kefford R, Dummer R, Kirkwood JM (2017). Adjuvant Dabrafenib plus Trametinib in stage III BRAF-mutated melanoma. New Engl J Med..

[11] Sanlorenzo M, Vujic I, Floris A, Novelli M, Gammaitoni L, Giraudo L, Macagno M, Leuci V, Rotolo R, Donini C, Basiricò M, Quaglino P, Fierro MT, Giordano S, Sibilia M, Carnevale-Schianca F, Aglietta M, Sangiolo D (2018). BRAF and MEK inhibitors increase PD-1-positive melanoma cells leading to a potential lymphocyte-independent synergism with anti–PD-1 antibody. Clin Cancer Res.

[12] Kunz M, Hölzel M (2017). The impact of melanoma genetics on treatment response and resistance in clinical and experimental studies. Cancer Metast Rev.

[13] Hu-Lieskovan S, Mok S, Homet Moreno B, Tsoi J, Robert L, Goedert L, Pinheiro EM, Koya RC, Graeber TG, Comin-Anduix B (2015). Improved antitumor activity of immunotherapy with BRAF and MEK inhibitors inBRAFV600E melanoma. Sci Transl Med.

[14] Valpione S, Campana LG (2016). Immunotherapy for advanced melanoma: future directions. Immunotherapy-UK..

[15] Axelrod ML, Johnson DB, Balko JM (2018). Emerging biomarkers for cancer immunotherapy in melanoma. Semin Cancer Biol.

[16] Dummer R, Ascierto PA, Nathan P, Robert C, Schadendorf D (2020). Rationale for immune checkpoint inhibitors plus targeted therapy in metastatic melanoma: a review. Jama Oncol..

[17] Effern M, Glodde N, Braun M, Liebing J, Boll HN, Yong M, Bawden E, Hinze D, van den Boorn-Konijnenberg D, Daoud M, Aymans P, Landsberg J, Smyth MJ, Flatz L, Tüting T, Bald T, Gebhardt T, Hölzel M (2020). Adoptive T cell therapy targeting different gene products reveals diverse and context-dependent immune evasion in melanoma. Immunity..

[18] Sha D, Jin Z, Budczies J, Kluck K, Stenzinger A, Sinicrope FA. Tumor Mutational Burden as a Predictive Biomarker in Solid Tumors. Cancer Discov. 2020;10(12):1808–25. 10.1158/2159-8290.CD-20-0522 PMC7710563 33139244

[19] Jardim DL, Goodman A, de Melo Gagliato D, Kurzrock R. The challenges of tumor mutational burden as an immunotherapy biomarker. Cancer cell. 2021;39(2):154–73. 10.1016/j.ccell.2020.10.001 PMC7878292 33125859

[20] Heydt C, Rehker J, Pappesch R, Buhl T, Ball M, Siebolts U, Haak A, Lohneis P, Büttner R, Hillmer AM *et al***.** Analysis of tumor mutational burden: correlation of five large gene panels with whole exome sequencing. Sci Rep-UK**.** 2020; 10(1). doi: 10.1038/s41598-020-68394-4. 10.1038/s41598-020-68394-4 PMC7347536 32647293

[21] Ajona D, Ortiz-Espinosa S, Moreno H, Lozano T, Pajares MJ, Agorreta J, Bértolo C, Lasarte JJ, Vicent S, Hoehlig K, Vater A, Lecanda F, Montuenga LM, Pio R (2017). A combined PD-1/C5a blockade synergistically protects against lung Cancer growth and metastasis. Cancer Discov.

[22] Razzak M (2013). Anti-PD-1 approaches—important steps forward in metastatic melanoma. Nat Rev Clin Oncol.

[23] A Set of Transcriptomic Changes Is Associated with Anti–PD-1 Resistance. Cancer Discov**.** 2016; 6(5):471–472.doi: 10.1158/2159-8290.CD-RW2016-057. 10.1158/2159-8290.CD-RW2016-057 27034380

[24] Cristescu R, Mogg R, Ayers M, Albright A, Murphy E, Yearley J, Sher X, Liu XQ, Lu H, Nebozhyn M (2018). Pan-tumor genomic biomarkers for PD-1 checkpoint blockade–based immunotherapy. Science..

[25] Hugo W, Zaretsky JM, Sun L, Song C, Moreno BH, Hu-Lieskovan S, Berent-Maoz B, Pang J, Chmielowski B, Cherry G, Seja E, Lomeli S, Kong X, Kelley MC, Sosman JA, Johnson DB, Ribas A, Lo RS (2016). Genomic and Transcriptomic features of response to anti-PD-1 therapy in metastatic melanoma. Cell..

[26] Mayakonda A, Lin D, Assenov Y, Plass C, Koeffler HP (2018). Maftools: efficient and comprehensive analysis of somatic variants in cancer. Genome Res.

[27] Ritchie ME, Phipson B, Wu D, Hu Y, Law CW, Shi W, Smyth GK (2015). limma powers differential expression analyses for RNA-sequencing and microarray studies. Nucleic Acids Res.

[28] Yu G, Wang L, Han Y, He Q (2012). clusterProfiler: an R package for comparing biological themes among gene clusters. OMICS.

[29] Subramanian A, Tamayo P, Mootha VK, Mukherjee S, Ebert BL, Gillette MA, Paulovich A, Pomeroy SL, Golub TR, Lander ES, Mesirov JP (2005). Gene set enrichment analysis: a knowledge-based approach for interpreting genome-wide expression profiles. Proc Natl Acad Sci.

[30] Liberzon A, Birger C, Thorvaldsdóttir H, Ghandi M, Mesirov JP, Tamayo P (2015). The molecular signatures database Hallmark gene set collection. Cell Syst.

[31] Bhattacharya S, Dunn P, Thomas CG, Smith B, Schaefer H, Chen J, Hu Z, Zalocusky KA, Shankar RD, Shen-Orr SS, Thomson E, Wiser J, Butte AJ (2018). ImmPort, toward repurposing of open access immunological assay data for translational and clinical research. SCI DATA.

[32] Li T, Fan J, Wang B, Traugh N, Chen Q, Liu JS, Li B, Liu XS (2017). TIMER: a web server for comprehensive analysis of tumor-infiltrating immune cells. Cancer Res.

[33] Newman AM, Steen CB, Liu CL, Gentles AJ, Chaudhuri AA, Scherer F, Khodadoust MS, Esfahani MS, Luca BA, Steiner D, Diehn M, Alizadeh AA (2019). Determining cell type abundance and expression from bulk tissues with digital cytometry. Nat Biotechnol.

[34] Couzin-Frankel J (2013). Cancer immunotherapy. Science..

[35] Van Allen EM, Miao D, Schilling B, Shukla SA, Blank C, Zimmer L, Sucker A, Hillen U, Geukes Foppen MH, Goldinger SM (2015). Genomic correlates of response to CTLA-4 blockade in metastatic melanoma. Science..

[36] Howitt BE, Shukla SA, Sholl LM, Ritterhouse LL, Watkins JC, Rodig S, Stover E, Strickland KC, D Andrea AD, Wu CJ *et al***.** Association of Polymerase e–mutated and microsatellite-instable endometrial cancers with Neoantigen load, number of tumor-infiltrating lymphocytes, and expression of PD-1 and PD-L1. Jama Oncol 2015; 1(9):1319, DOI: 10.1001/jamaoncol.2015.2151. 10.1001/jamaoncol.2015.2151 26181000

[37] Chan TA, Wolchok JD, Snyder A (2015). Genetic basis for clinical response to CTLA-4 blockade in melanoma. New Engl J Med..

[38] Rizvi NA, Hellmann MD, Snyder A, Kvistborg P, Makarov V, Havel JJ, Lee W, Yuan J, Wong P, Ho TS, Miller ML, Rekhtman N, Moreira AL, Ibrahim F, Bruggeman C, Gasmi B, Zappasodi R, Maeda Y, Sander C, Garon EB, Merghoub T, Wolchok JD, Schumacher TN, Chan TA (2015). Mutational landscape determines sensitivity to PD-1 blockade in non–small cell lung cancer. Science..

[39] Hellmann MD, Ciuleanu T, Pluzanski A, Lee JS, Otterson GA, Audigier-Valette C, Minenza E, Linardou H, Burgers S, Salman P (2018). Nivolumab plus Ipilimumab in lung Cancer with a high tumor mutational burden. New Engl J Med.

[40] Rosenthal R, Cadieux EL, Salgado R, Bakir MA, Moore DA, Hiley CT, Lund T, Tanić M, Reading JL, Joshi K (2019). Neoantigen-directed immune escape in lung cancer evolution. Nature..

[41] Desrichard A, Kuo F, Chowell D, Lee K, Riaz N, Wong RJ, Chan TA, Morris LGT (2018). Tobacco smoking-associated alterations in the immune microenvironment of squamous cell carcinomas. J Natl Cancer Institute.

[42] Fredriksson NJ, Elliott K, Filges S, Van den Eynden J, Ståhlberg A, Larsson E (2017). Recurrent promoter mutations in melanoma are defined by an extended context-specific mutational signature. PLoS Genet.

[43] Schreiber RD, Old LJ, Smyth MJ (2011). Cancer Immunoediting: integrating Immunity's roles in Cancer suppression and promotion. SCIENCE..

[44] Rieckmann JC, Geiger R, Hornburg D, Wolf T, Kveler K, Jarrossay D, Sallusto F, Shen-Orr SS, Lanzavecchia A, Mann M, Meissner F (2017). Social network architecture of human immune cells unveiled by quantitative proteomics. Nat Immunol.

[45] Cao Y, Wang X, Jin T, Tian Y, Dai C, Widarma C, Song R, Xu F (2020). Immune checkpoint molecules in natural killer cells as potential targets for cancer immunotherapy. Signal Transduction Targeted Therapy.

[46] Coleman E, Ko C, Dai F, Tomayko MM, Kluger H, Leventhal JS (2019). Inflammatory eruptions associated with immune checkpoint inhibitor therapy: a single-institution retrospective analysis with stratification of reactions by toxicity and implications for management. J Am Acad Dermatol.

[47] Lynes J, Jackson S, Sanchez V, Dominah G, Wang X, Kuek A, Hayes CP, Benzo S, Scott GC, Chittiboina P, Zaghloul KA, Park DM, Wu J, Hourigan CS, Giles AJ, Wu T, Maric D, Chen J, Quezado M, Heiss JD, Gilbert MR, Nduom EK (2019). Cytokine microdialysis for real-time immune monitoring in glioblastoma patients undergoing checkpoint blockade. Neurosurgery..

[48] Routy B, Le Chatelier E, Derosa L, Duong CPM, Alou MT, Daillère R, Fluckiger A, Messaoudene M, Rauber C, Roberti MP (2018). Gut microbiome influences efficacy of PD-1–based immunotherapy against epithelial tumors. Science..

[49] Schumacher TN, Schreiber RD (2015). Neoantigens in cancer immunotherapy. Science..

