# Supplementary figures and images for: Gene expression and immune infiltration in melanoma patients with different mutation burden
Source: BMC Cancer. 2021 Apr 9;21:379. doi: 10.1186/s12885-021-08083-1 (PMC8034108; doi:10.1186/s12885-021-08083-1)

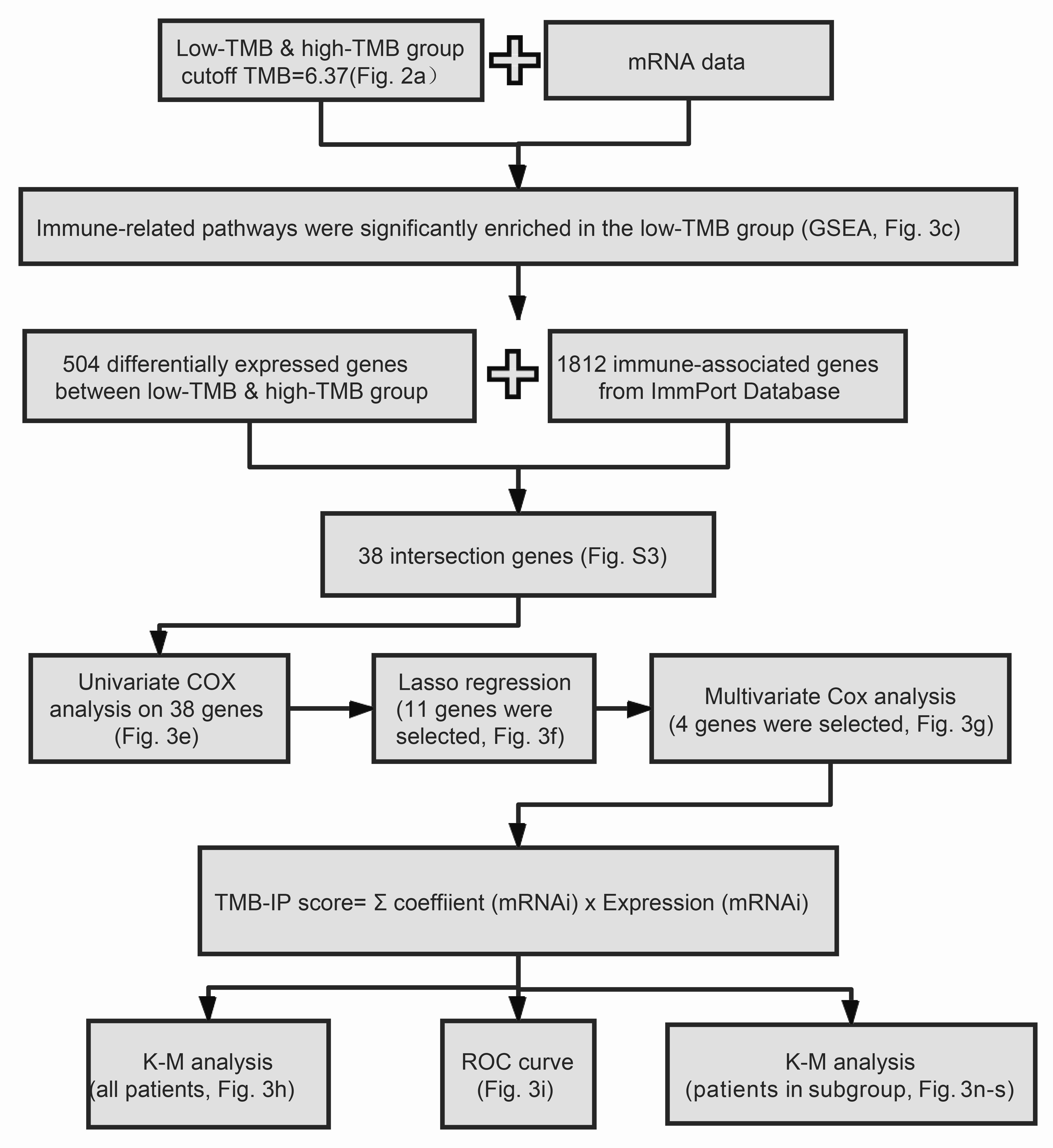

Supplement: Supplementary file 1 — Additional file 1: Figure S1. Workflow of this study. [file 12885_2021_8083_MOESM1_ESM.tif]

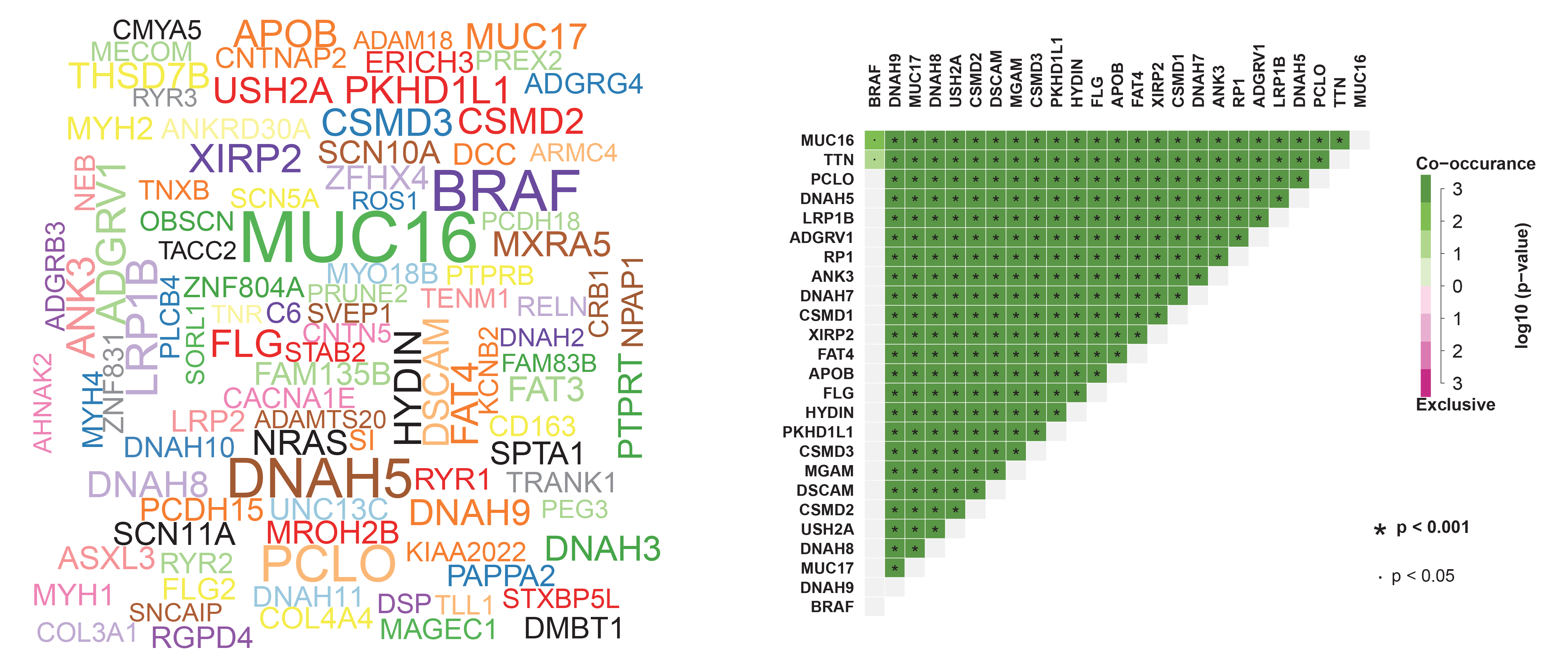

Supplement: Supplementary file 2 — Additional file 2: Figure S2. The genecloud plot and the coincidences&exclusive relationship plot among the mutated genes. [file 12885_2021_8083_MOESM2_ESM.tif]

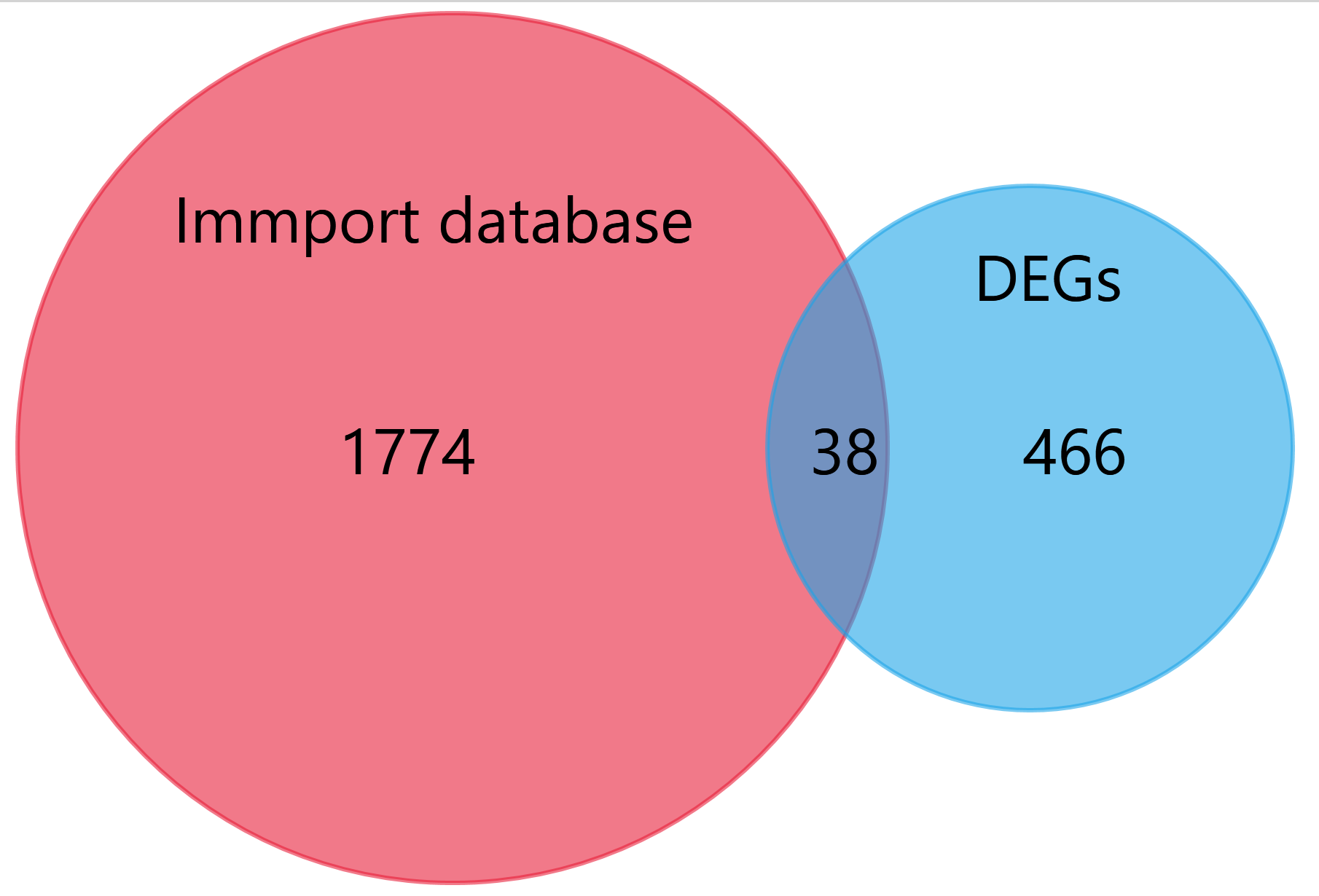

Supplement: Supplementary file 3 — Additional file 3: Figure S3. Venn analysis of 1812 immune genes and 504 DEGs. [file 12885_2021_8083_MOESM3_ESM.tif]

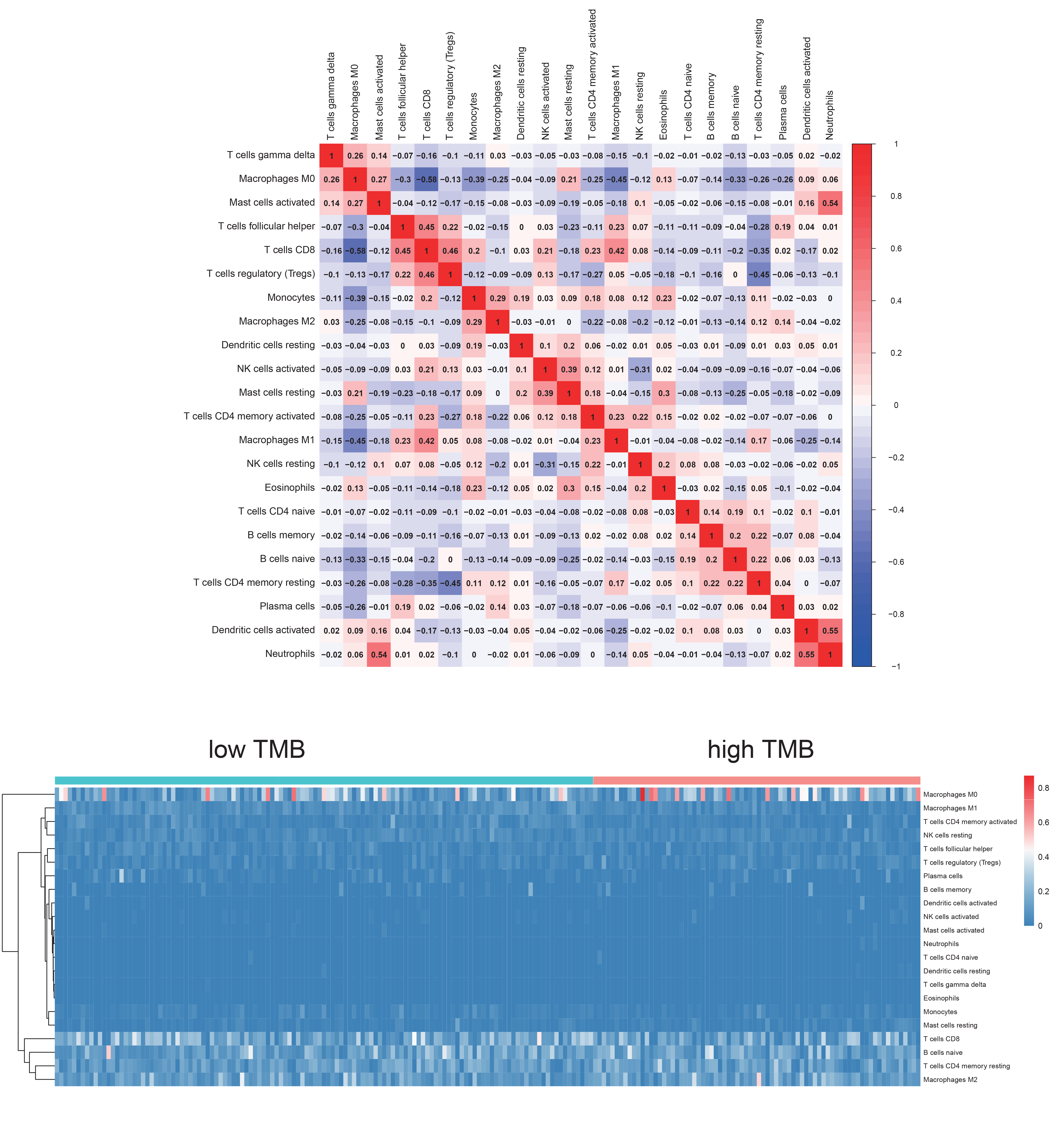

Supplement: Supplementary file 4 — Additional file 4: Figure S4. Correlation and distribution analysis of the infiltration degree of 22 immune cells in the high-TMB and low-TMB groups. [file 12885_2021_8083_MOESM4_ESM.tif]
